# Anti-Infective Effect of Adhesive Probiotic *Lactobacillus* in Fish is Correlated With Their Spatial Distribution in the Intestinal Tissue

**DOI:** 10.1038/s41598-017-13466-1

**Published:** 2017-10-16

**Authors:** Suxu He, Chao Ran, Chubin Qin, Shuning Li, Hongling Zhang, Willem M. de Vos, Einar Ringø, Zhigang Zhou

**Affiliations:** 1 Key Laboratory for Feed Biotechnology of the Ministry of Agriculture, Feed Research Institute, Chinese Academy of Agricultural Sciences, Beijing, 100081 P. R. China; 2 Laboratory of Microbiology, Wageningen University, Wageningen, Netherlands and Departments of Bacteriology & Immunology and Veterinary Biosciences, University of Helsinki, Helsinki, Finland; 3 Norwegian College of Fishery Science, Faculty of Bioscience, Fisheries and Economics, University of Tromsø, NO-9037 Tromsø, Norway

## Abstract

In this study, we tested the distribution of 49 *Lactobacillus* strains in the mucus and mucosa of the intestine tissue of zebrafish. We observed a progressive change in the spatial distribution of *Lactobacillus* strains, and suggested a division of the strains into three classes: mucus type (>70% in mucus), mucosa type (>70% in mucosa) and hybrid type (others). The hybrid type strains were more efficient in protection of zebrafish against *Aeromonas hydrophila* infection. Three strains representing different distribution types (JCM1149, CGMCC1.2028, and JCM 20300) were selected. The mucosa type strain JCM1149 induced higher intestinal expression of inflammatory cytokines and *Hsp70* than the other strains. Furthermore, we used *L. rhamnosus* GG and its mutant (PB22) lacking SpaCBA pili to investigate the influence of pili on spatial distribution. LGG showed a mucosa type distribution, while PB22 revealed a hybrid distribution and the disease protection was accordingly improved. The different protection ability between LGG and PB22 did not involve the intestinal microbiota, however, LGG induced injury to the mucosa of zebrafish. Collectively, the disease protection activity of *Lactobacillus* in zebrafish is correlated with their spatial distribution in the intestinal tissue, with strains showing a balanced distribution (hybrid type) more efficient in protection.

## Introduction

*Lactobacillus* are used in many biotechnological applications and many are marketed as probiotics because of their health-promoting properties^1–4^. Several strains of *Lactobacillus* have been developed for use in fish and other animals, including *L. acidophilus*, *L. lactis*, *L. plantarum*, and *L. rhamnosus* ^5^. Moreover, some of the lactobacilli isolated from the human intestinal tract have shown good probiotic efficacy in animals. One such example is *Lactobacillus rhamnosus* strain GG^6^. In aquaculture, one of the main benefits associated with *Lactobacillus* application is the protection of the fish against pathogen infection, as reported in many studies^7–9^.

Adhesion is considered a desirable feature for a probiotic strain as it can promote the gut residence of probiotics as well as their interaction with host epithelial and immune cells^1,2,10,11^. Previous study in zebrafish revealed a key role of adhesion in protection activity of probiotic bacteria against pathogen infection^12^. Consistently, in our previous study, highly-adhesive *Lactobacillus* strains were more efficient in protection of zebrafish against *A. hydrophila* infection, indicating the importance of adhesion in the disease protection effect of *Lactobacillus* ^5^.

The intestinal mucosa consists of one or more layers of epithelial cells overlaying a layer of loose connective tissue^13^. The mucosa is covered with a protective mucus layer^14^. The adhesion of *Lactobacillus* strains to mucus and intestinal epithelial cells has been reported in many studies^12,15–17^. *Lactobacillus* cell surface components (the mucus-binding (MUB) protein, LPXTG and pili) play important roles in the adherence of lactobacilli to the intestine, as well as host interactions^11,12,16,17^. *L. rhamnosus* GG adhere to gut mucosa through SpaC, a pili component protein which could also induce epithelial generation of ROS and extracellular signal-regulated kinase/mitogen-activated protein kinase (ERK/MAPK) signaling in enterocytes to benefit host^17^.

Studies regarding the intestinal adherence of *Lactobacillus in vivo* mostly address the overall binding of *Lactobacillus* to the inner surface of intestine^6,17^. However, the spatial distribution of the bound cells in the intestinal tissue has been rarely investigated. In a pioneering study it has been reported that *Bacteroides fragilis* penetrates the mucus and resides deep within the crypt channels in the colon of mice^18^. Previously, we found that *Lactobacillus* strains with overall good intestinal adherence properties in fish showed differential relative distribution in mucosa and mucus^19^. In the current study, we further investigated the intestinal spatial distribution property of a collection of *Lactobacillus* strains in zebrafish. Three types of spatial distribution were observed among the strains. Intriguingly, the spatial distribution property of *Lactobacillus* strains was correlated with their disease protection ability in zebrafish. Moreover, using LGG as the model strain, we revealed that the SpaCBA pili is a determinant of the spatial distribution, and the mechanism underlying the differential disease protection activity of strains with different spatial distribution was investigated.

## Results

### Protection activity of *Lactobacillus* strains is correlated with their spatial distribution in the intestine

**Figure 1:**
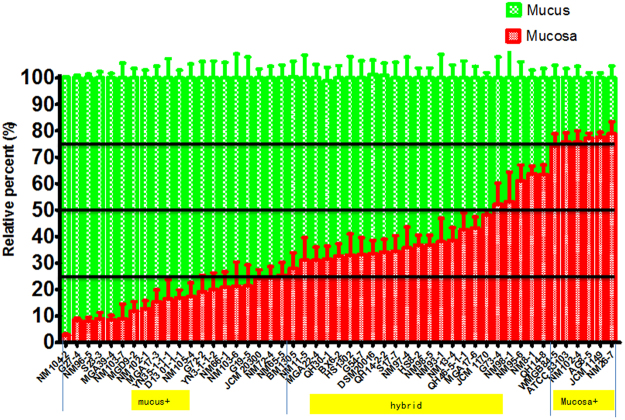
The spatial distribution of 49 *Lactobacillus* strains in zebrafish gut. The fish were immersed in *Lactobacillus*-inoculated water for 14 days. n = 8.

**Figure 2:**
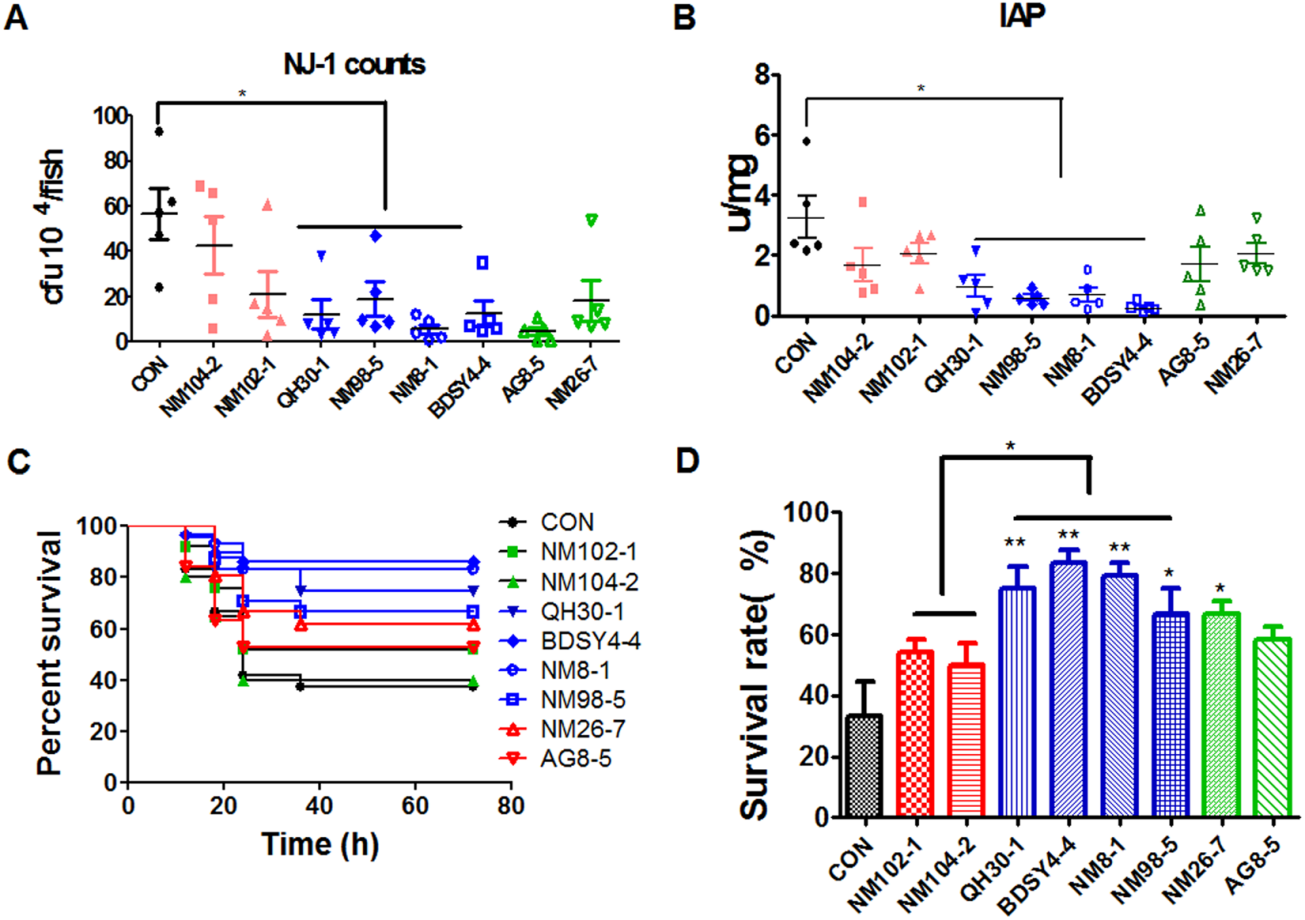
Protective ability of selected *Lactobacillus* strains representing different spatial distribution types. (**A**) Abundance of *A. hydrophila* NJ-1 in the gut of zebrafish 24 h after challenge (**B**) IAP activity of zebrafish 24 h after challenge with *A. hydrophila* NJ-1. (**C**) Cumulative survival of zebrafish after infection. (**D**) Final survival of zebrafish. All data are presented as mean ± SEM, ^*^ *P* < 0.05, ^**^ *P* < 0.01.

The spatial distribution of 49 *Lactobacillus* strains (Table S3) in the intestine of zebrafish were tested. Interestingly, the distribution of the 49 strains showed a progressive change, from 89.6% mucosa/10.4% mucus for NM-26–7 to 5.3% mucosa/94.7% mucus for NM104–2 (Fig. 1). Based on the spatial distribution, we categorized the *Lactobacillus* strains into three classes: strains with more than 70% cells located in the mucus zone are mucus type; strains with more than 70% cells in the mucosa zone are mucosa type; other strains showing a relative balanced distribution are considered as hybrid type (Fig. 1). We further tested infection protection activity of *Lactobacillus* strains from different spatial distribution classes. Eight *La*ctobacillus strains representing different adhering types were randomly selected. Among them, NM104-2 and NM102-1 are mucus type strains; QH30–1, NM98-5, NM8-1 and BDSY 4-4 belong to hybrid type; AG8-5 and NM26-7 are mucosa type ones. Zebrafish were immersed in water inoculated with one of the 8 strains and challenged by *A. hydrophila* NJ-1. Compared with control group, zebrafish treated with hybrid type strains exhibited lower NJ-1counts in the gut (Fig. 2A), and lower IAP activity post challenge (Fig. 2B). Accordingly, treatment by hybrid type strains led to higher survival rate of zebrafish compared with control group, indicating a higher protection activity (Fig. 2C,D). Therefore, the disease protection activity of *Lactobacillus* strains in zebrafish is correlated with their spatial distribution in the intestine, and strains showing a balanced distribution (hybrid type strains) are more efficient in disease protection compared with either mucus or mucosa type strains.

### The phenotypes of three well-studied probiotic *Lactobacillus* strains in zebrafish

**Figure 3:**
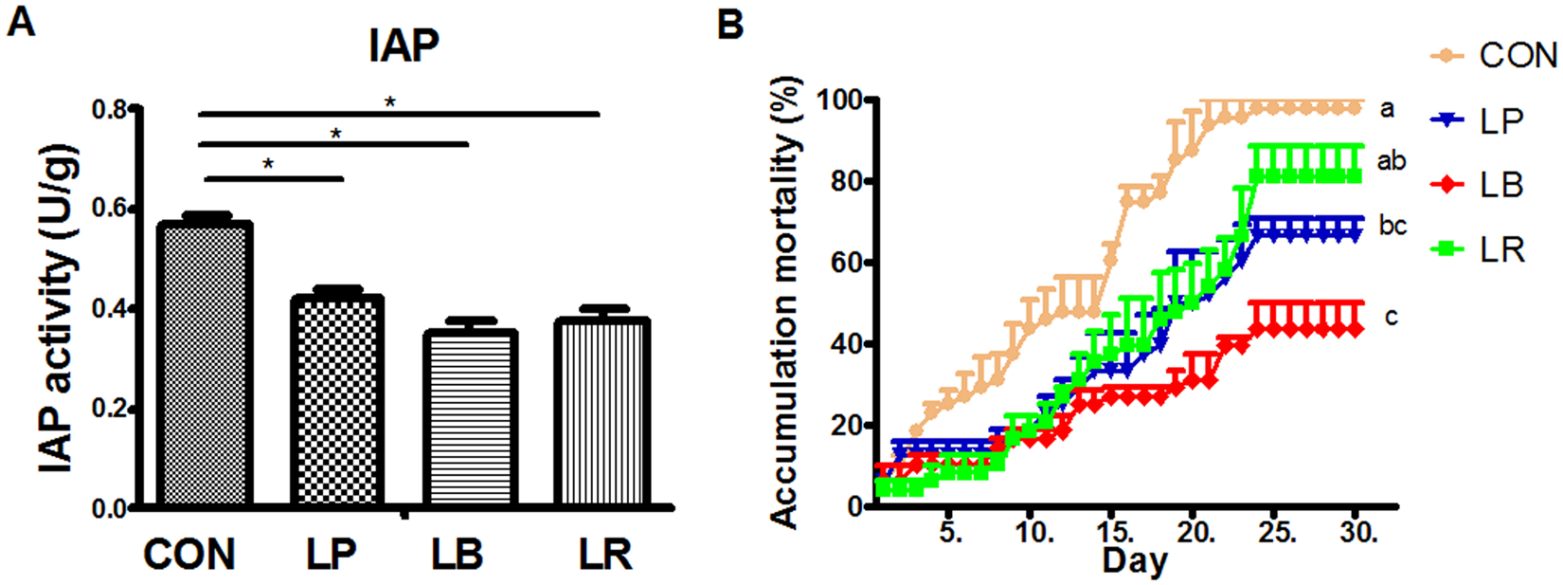
Protection of zebrafish against *A. hydrophila* NJ-1 by the three *Lactobacillus* strains after 14 days immersion treatment. (**A**) Intestinal alkaline phosphatase (IAP) activity after *A. hydrophila* NJ-1 infection for 24 h. (**B**) Cumulative mortality of zebrafish after *A. hydrophila* NJ-1 infection. Data were presented as mean** ± **SEM. ^*^P < 0.05, ^**^P < 0.01. Means sharing a common letter (a,b,c) were not significantly different (*P* > 0.05). LP, *L. plantarum* JCM1149; LB, *L. brevis* CGMCC1.2028; LR, *L. rhamnosus* JCM 20300; CON, control.

From the 49 strains, three well-studied strains representing different spatial distribution types were selected for further investigation: *L. plantarum* subsp. *plantarum* JCM1149 (mucosa type, LP), *L. brevis* CGMCC1.2028 (hybrid type, LB) and *L. rhamnosus* JCM 20300 (mucus type, LR). The mucus/mucosa distribution for LR, LB and LP was 75%/25%, 65%/35%, and 20%/80%, respectively (Fig. 1, Figs S3, S4). The protection activity of each strain against *A. hydrophila* infection of zebrafish was also tested. Zebrafish were immersed with *Lactobacillus* at 10^7^ cfu/ml (Fig. S1) for 14 days and challenged by NJ-1. All the three strains decreased the cumulative mortality of zebrafish compared with control group, while LB showed the highest protection (Fig. 3B). A similar trend was observed for IAP activity post challenge (Fig. 3A). These results confirmed that the hybrid type strain is more efficient in protection of zebrafish against infection.

*In vitro* test showed no difference in antagonistic activity of the three *Lactobacillus* strains against NJ-1, indicating that the differential protection activity was not due to antagonism (Fig. S1A). Notably, the three stains showed different releasing dynamics from the intestine of zebrafish after cessation of administration. LP showed a strong retaining capacity in mucosa, while LB exhibited good retaining in both mucosa and mucus. In contrast, LR disappeared quickly in both mucus and mucosa of fish (Fig. S5).

### The effect of the *Lactobacillus* strains on the intestinal microbiota and expression of immunity-related genes in zebrafish

**Figure 4:**
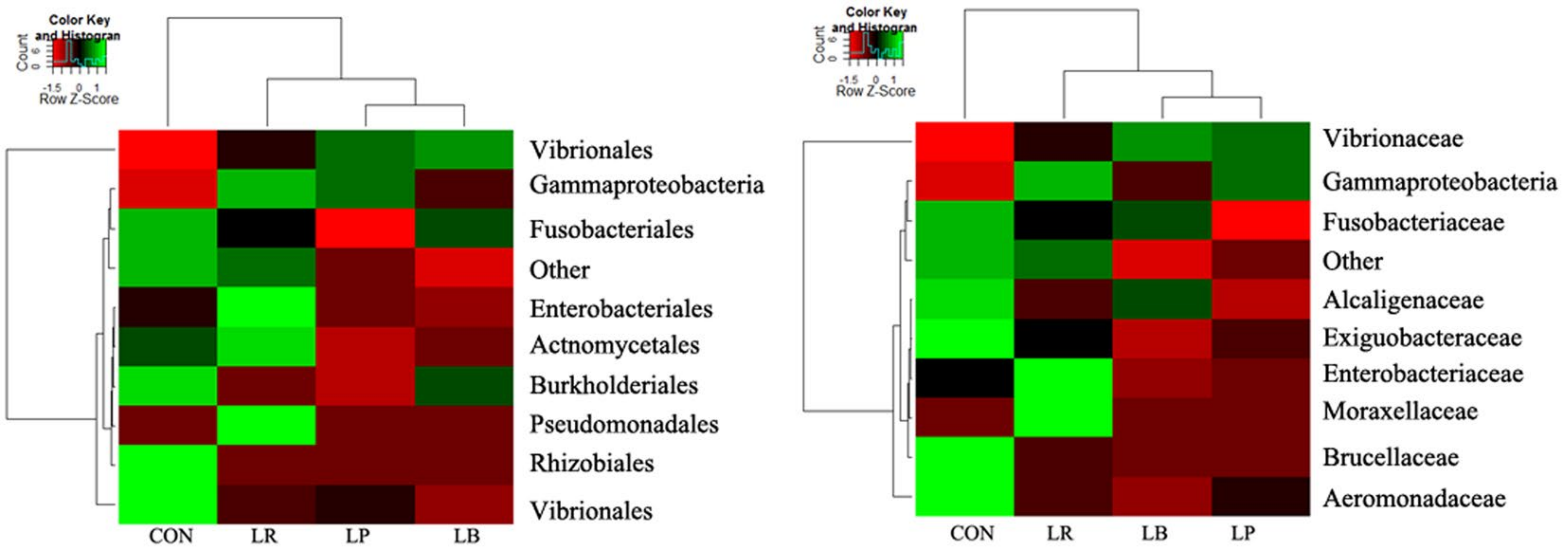
Effect of the three *Lactobacillus* strains treatment on the intestinal microbiota of zebrafish. Heatmap of the 10 most abundant family (**A**) and genus (**B**) in the intestinal microbiota of zebrafish from different treatments. LP, *L. plantarum* JCM1149; LB, *L. brevis* CGMCC1.2028; LR, *L*. *rhamnosus* JCM 20300; CON, control.

The colonization of each of the three *Lactobacillus* strains induced alteration in the intestinal microbiota of zebrafish compared with the control (Fig. 4). Notably, the magnitude of microbiota change relative to the control was larger in LP- and LB-treated fish compared with LR-treated counterparts (Fig. 4).

**Figure 5:**
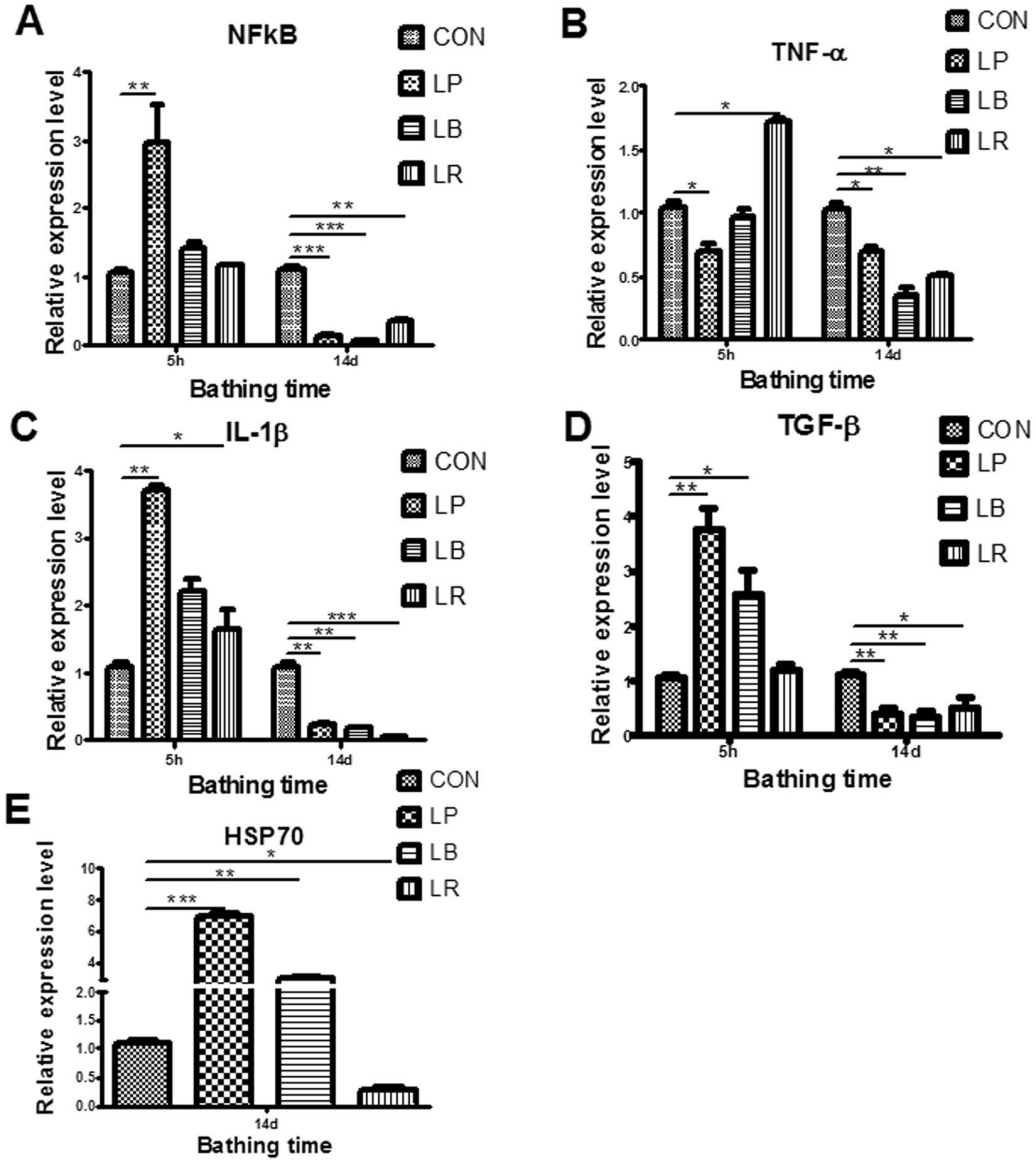
Effect of the three *Lactobacillus* strains on the intestinal expression of immunity-related genes in zebrafish. (**A**–**D**) nuclear factor kappa B (NF-kB), tumor necrosis factor (TNF)-α, interleukin IL-1β, and transforming growth factor-β (TGF-β) expression levels in gut of zebrafish after *Lactobacillus* immersion for 5 h and 14 days; (**E**) Hsp70 expression levels in gut of zebrafish after *Lactobacillus* immersion for 14 days. All data are presented as mean ± SEM, ^*^ *P* < 0.05, ^**^ *P* < 0.01, ^***^ *P* < 0.001. LP, *L. plantarum* JCM1149; LB, *L. brevis* CGMCC1.2028; LR, *L. rhamnosus* JCM 20300; CON, control.

To study the correlation between spatial distribution and immunomodulation of the *Lactobacillus* strains, we determined the expression of NF-κB, cytokines, and *hsp70* (Fig. 5). The *Lactobacillus* treatments for 5 h increased the expression levels of NF-κB, TNF-α, IL-1β and TGF-β at the initial stage. Notably, the gene expression was significantly higher in LP-treated fish than those receiving LB or LR (Fig. 5). An increased incubation time with *Lactobacillus* resulted in significantly lower levels of cytokines. However, a higher *hsp70* expression level was observed in LP-treated fish compared with fish treated with LB, LR, and control.

### LGG mutant lacking SpaCBA pili showed changed spatial distribution and protection activity

**Figure 6:**
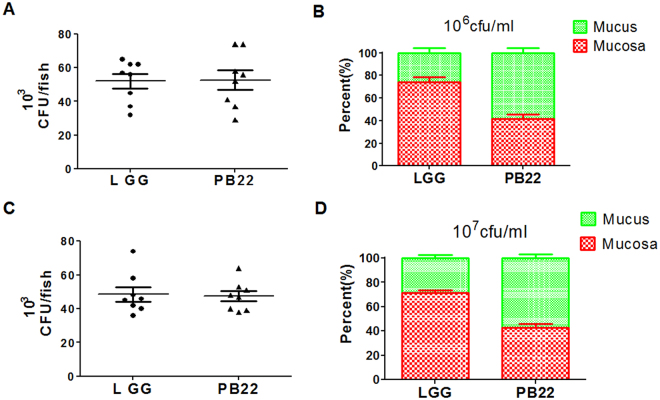
Spatial distribution of LGG and LGG mutant PB22 in the gut of zebrafish. (**A**) The overall colonization level of LGG or PB22 in fish gut at 10^6^ cfu/ml immersion. (**B**) Spatial distribution of LGG or PB22 at 10^6^ cfu/ml immersion. (**C**) The overall colonization level of LGG or PB22 in fish gut at 10^7^ cfu/ml immersion. (**D**) Spatial distribution of LGG or PB22 at 10^7^ cfu/ml immersion. LGG, *L. rhamnosus* GG; PB22, LGG mutant PB22. PB22 is pilus deficient as it has lost the pilus island and flanking sequences (75 kb DNA) and has 51 other SNPs.

**Figure 7:**
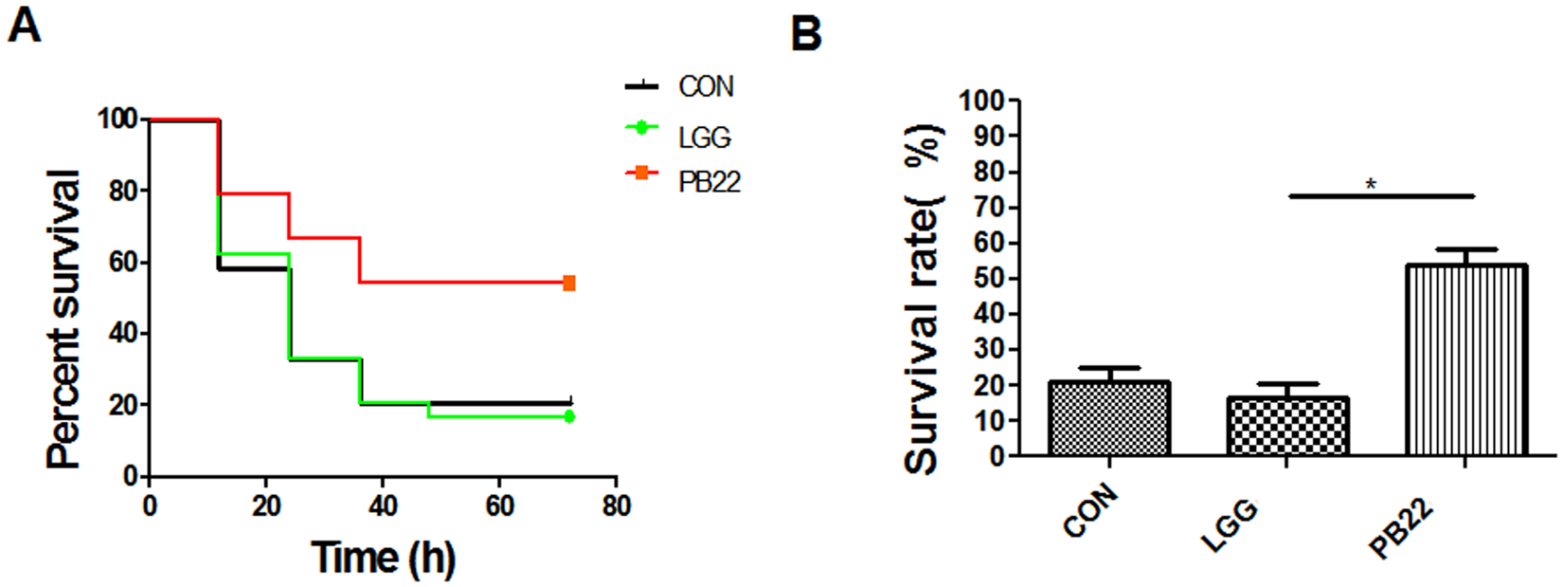
Protection ability of LGG and mutant PB22 in adult zebrafish. Zebrafish were immersed for 14 days in water inoculated with each strain at 10^7^ cfu/ml and were challenged with *A. hydrophila* NJ-1. (**A**) Cumulative survival of zebrafish after infection, (**B**) The final survival of zebrafish after infection. Data were presented as mean ± SEM. ^*^P < 0.05, ^**^P < 0.01. CON, control; LGG, *L. rhamnosus* GG; PB22, LGG mutant PB22.

We further used the well-studied probiotic strain *L.rhamnosus* GG (LGG) for a mechanistic investigation. In accordance with previous studies, LGG showed good adhesion to the intestinal inner surface of zebrafish. Moreover, LGG showed a mucosa type distribution, with the majority of LGG cells bound in the mucosa zone (Fig. 1). Next, the isogenic pili-deficient LGG mutant strain (PB22)^19^ was used to investigate whether the well-studied mucus-binding pili of LGG influence its spatial distribution. The total colonization level on the inner intestinal surface was not different for PB22 compared with LGG (Fig. 6). However, the spatial distribution of PB22 was significantly changed, with a reduction of cells bound to the mucosa and an according increase in the mucus bound cells (Fig. 6). To investigate whether the altered spatial distribution influence the protection ability against pathogen, zebrafish were immersed with the wild type LGG and the mutant PB22 at 10^7^ cfu/ml as described above and challenged with *A. hydrophila*. The results showed that PB22 showed significantly higher protection activity than LGG wild type (Fig. 7).

### The difference in protection activity between LGG and PB22 does not involve intestinal microbiota

**Figure 8:**
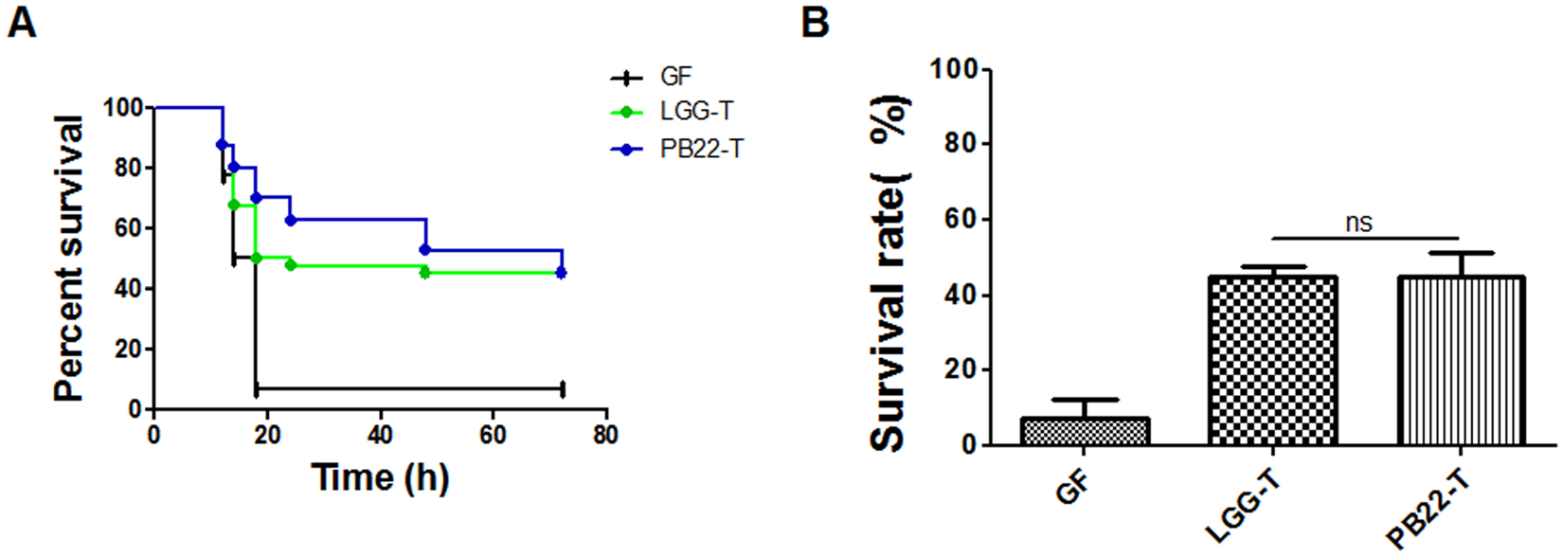
Protective effects of the gut microbiotas transplanted from adult zebrafish in recipient GF zebrafish against *A.hydrophila* NJ-1 infection. (**A**) Cumulative survival for different groups. (**B**) The final survival of GF zebrafish colonized with gut microbiotas for 3 days. All values are presented as mean ± SEM, ^*^ *P* < 0.05. GF, germ-free zebrafish; LGG, *L. rhamnosus* GG; PB22, LGG mutant PB22.

**Figure 9:**
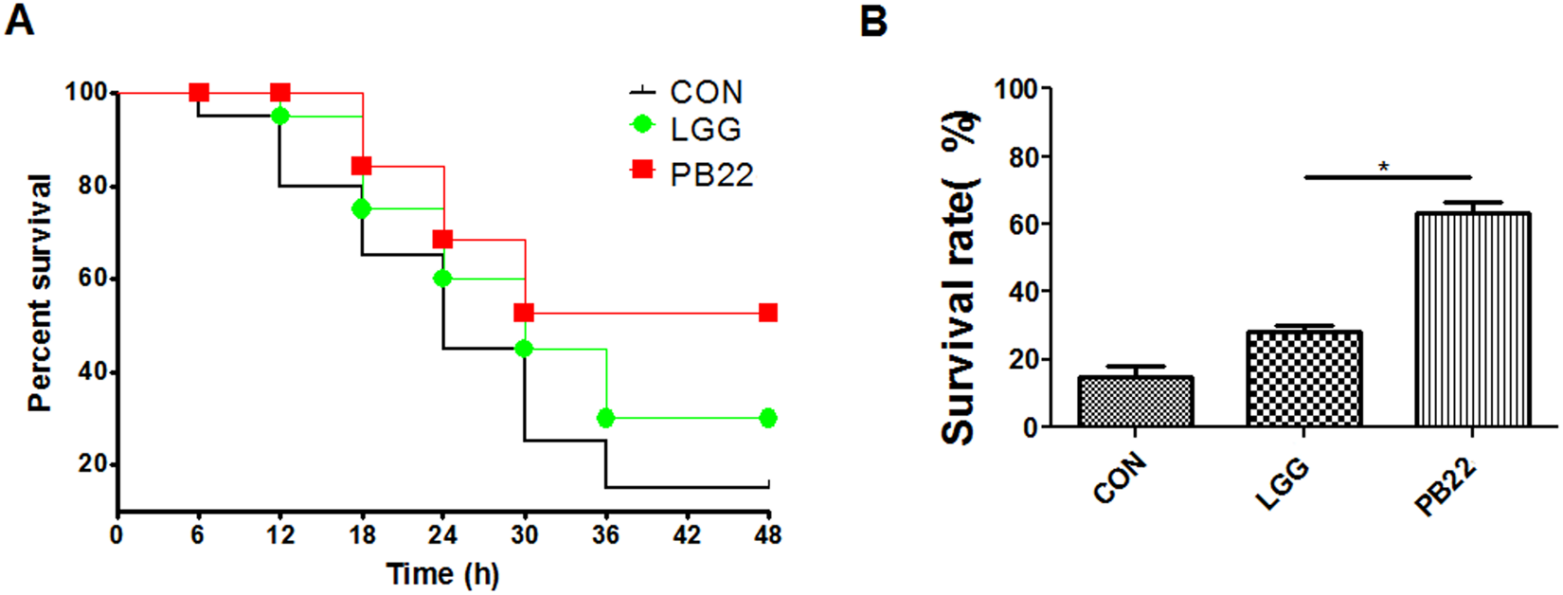
Protection ability of LGG and PB22 in GF zebrafish larvae. GF zebrafish at 3 dpf were immersed in water inoculated with each strain (10^7^ cfu/ml) and were challenged with NJ-1 at 6 dpf. (**A**) Cumulative survival of zebrafish larvae after infection. (**B**) The final survival of zebrafish larvae after infection. Data were presented as mean ± SEM. ^*^P < 0.05. CON, control; LGG, *L. rhamnosus* GG; PB22, LGG mutant PB22.

To investigate whether the correlation between spatial distribution and protection activity of *Lactobacillus* in fish involves the intestinal microbiota, GF zebrafish were colonized with the microbiotas from zebrafish that have been treated by LGG or PB22, and were challenged by *A. hydrophila* NJ-1 (Fig. 8). No survival differences were observed between GF zebrafish colonized with LGG- and PB22-microbiota at each inoculation concentration of the microbiota, indicating that the difference in protection activity between LGG and PB22 was not mediated by the microbiota of zebrafish. Moreover, GF zebrafish directly treated with PB22 showed a higher survival compared with LGG treated counterparts post *Aeromonas* infection (Fig. 9), further supporting that the spatial distribution-induced difference in protection activity is mediated by direct effect of *Lactobacillus* strains and does not likely to involve intestinal microbiota.

### LGG induced injury to the intestinal mucosa of zebrafish

**Figure 10:**
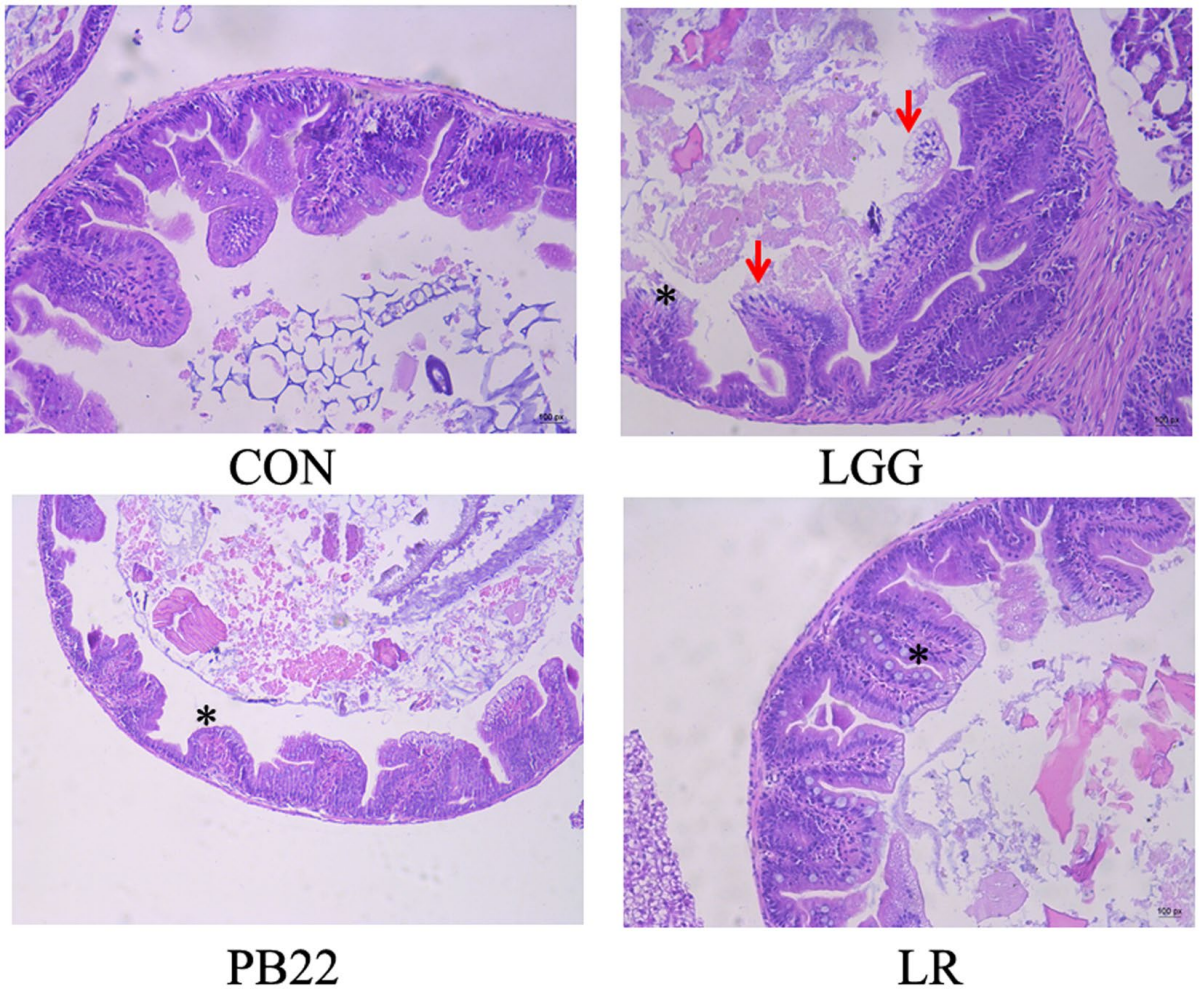
Histology (H&E staining) of intestine of zebrafish after immersion treatment with *Lactobacillus* strains for 14 days. CON, control; LGG, *L. rhamnosus* GG; PB22, LGG mutant PB22; LR, *L. rhamnosus* JCM 20300 Asterisk, edema and floating of the intestinal mucous membrane. Arrow, degeneration and focal necrosis of intestinal villus.

We next investigated the effect of *Lactobacillus* colonization on the mucosa tissue of zebrafish (Fig. 10). Both LGG and mutant PB22 were included in the test. Due to the lack of an available LGG mutant with mucus type distribution, *L. rhamnosus* JCM20300 (LR) was also included to represent the mucus type strain. Zebrafish intestines without inoculation of *Lactobacillus* strains displayed normal histological structure and cells in intestinal epithelium were arranged orderly. Significant pathological changes were observed in the anterior intestine of zebrafish treated with 10^7^ CFU/ml LGG for 14 days, including intestinal villi edema and congestion with slight infiltration of inflammatory cells (asterisk), vacuolar degeneration, necrosis and shedding of the epithelium cells (arrow). Slight edema and little inflammatory cells infiltration (asterisk) were found in the intestinal tissue of PB22 and LR treated zebrafish, accompanied by vacuolar degeneration of epithelial cells (Fig. 10).

### Mixture of mucus and mucosa type strains confers protection activity comparable to hybrid type strain

**Figure 11:**
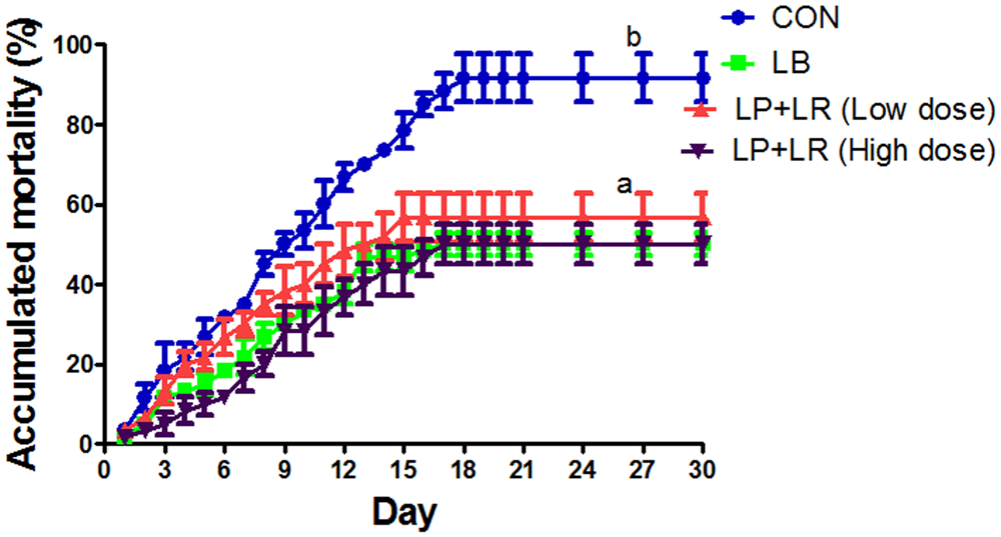
Cumulative mortality of NJ-1 challenged zebrafish after immersion in LB or equal mixture of LP and LR. Zebrafish were immersed with LB (10^7^ cfu/ml), LP + LR (0.5 × 10^7^ cfu/ml LP, 0.5 × 10^7^ cfu/ml LR; low dose), LP + LR (10^7^ cfu/ml LP, 10^7^ cfu/ml LR; high dose) for 14 days, then challenge with 10^8^ cfu/ml *A*. *hydrophila* NJ-1. Data marked with different letters (**a,b**) were significantly different (*P* < 0.05).

To test whether mixture of mucus and mucosa type strains obtain similar protection activity as hybrid type strain, zebrafish were immersed in water inoculated with equal mixture of LP and LR for 14 d and then challenged with NJ-1. Both mixture and LB treatments increased the survival of zebrafish compared with control (*P* < 0.05), and the survival of mixture- and LB-treated zebrafish was similar(Fig. 11), indicating comparable protection activity between the mixture and the hybrid type strain.

## Discussion

In a previous study, we observed different spatial distribution of 5 adherent *Lactobacillus* strains in the intestine of tilapia^19^. Here, we further investigated the spatial distribution of a larger number of adherent *Lactobacillus* strains in zebrafish, and evaluated the relationship between spatial distribution and the disease protection effect. Intriguingly, we observed a progressive change in the spatial distribution of the tested *Lactobacillus* strains, from typical mucus type to typical mucosa type strains. Moreover, the spatial distribution of *Lactobacillus* was correlated with disease protection activity, with strains showing a balanced mucus-mucosa distribution (hybrid type) more efficient in protection. To our knowledge, this is the first report on the correlation between intestinal spatial distribution and anti-infective effect of probiotics.

Mucins are heavily glycosylated, and many species of microbes have the ability to attach to these glycans and use them as a nutritional source^18^. Consistent with this, there is growing evidence that host-secreted glycans are important for the spatial distribution of commensal microbes^18,19^. In this regard, the relationship between spatial distribution of *Lactobacillus* strains and their capacity in glycans degradation deserves further investigation. The GI mucosal barrier is made up of epithelial and immune cells^16^^.^ *Lactobacilli* are able to modulate immune responses of the host by interaction with the GIT mucosa^8,11,16^. In our study, strains from the three distribution types induced the production of IL-1β, NF-κB, TGF-β and TNF-α at the initial stage. However, the expression of these genes was significantly lower than control at day 14 (Fig. 5). Mucosa type strain LP induced a higher *Hsp70* expression level until 14 days. This may be explained by the high levels of LP in mucosa, which may induce host stress response.

The three types of colonization in GI showed a different releasing dynamics after cessation of administration. LR was released quickly in both the mucus and mucosa. This is consistent with the relatively low protection efficiency against pathogen by LR, as the LR-colonized niches would be vacant after releasing, leading to a weak resilience against the invasion of pathogen. Similarly, a correlation of spatial distribution with colonization stability was reported in *B. fragilis*, with the mutant deficient to penetrate the colonic mucus showing less resilient colonization in the gut of mice^18^. However, the releasing dynamics cannot explain the lower protection of LP (mucosa type) relative to LB (hybrid type), as LP retains as well in mucus and shows an even stronger retaining in mucosa compared with LB. Therefore, we further investigated the mechanisms underlying the disease protection difference between mucosa and hybrid types of *Lactobacillus*.

LGG is one of the most thoroughly studied probiotic strains^6,15^. We took advantage of an available LGG mutant PB22, which lacks the SpaCBA pili that are important in colonization of LGG in the intestine of human and mice^15^. Surprisingly, mutant strain PB22 showed the same level of overall colonization in the intestine of zebrafish compared with the wildtype, while the spatial distribution of mutant PB22 changed to hybrid type from the mucosa type of the wildtype strain. Moreover, PB22 showed higher protection against pathogen infection compared with LGG, which is consistent with the observed pattern using different *Lactobacillus* species/strains. This suggested that the disease protection of *Lactobacillus* is highly correlated with spatial distribution, and does not involve other factors that are species- or strain-specific. Notably, mutant PB22 has 51 other SNPs besides pili deficiency. To confirm the phenotypes of PB22 was attributed to the lack of pili, the property of another LGG mutant PB12 was also investigated. PB12 is also pili deficient due to a mutation of the *srtC1* gene and it has a total of 24 other SNPs. The overall colonization, spatial distribution, as well as disease protection activity of both mutant strains accorded well with each other (Fig. S6), which indicated that the observed phenotypes of PB22 were due to pili deficiency.

The gut microbiota has been recognized as closely related with host health and immunity^20,21^. We have previously shown that the effect of essential oil components on the immunity of tilapia was a combined action of both direct effect of the compounds and the microbiota-mediated effect^22^. Considering the differential alterations of microbiota by the *Lactobacillus* spp., we firstly asked whether the difference in protection activity between mucosa type and hybrid type strains involves microbiota-mediated effect. The GF zebrafish colonized with LGG- or PB22-altered microbiota showed similar mortality post *A. hydrophila* challenge, indicating that the difference in protection between LGG and PB22 was not mediated by the microbiota of zebrafish. This is further confirmed by direct administration of LGG and PB22 to GF zebrafish. Therefore, the mechanism underlying the disease protection difference of hybrid and mucosa type strains does not involve a microbiota-mediated effect, and other effects directly associated with the host tissue interaction of *Lactobacillus* strains are playing the key role.

Considering the key role of direct interaction of *Lactobacillus* spp. with host tissue, we then observed the effect of *Lactobacillus* colonization on the intestinal mucosa of zebrafish. Surprisingly, LGG induced injury to the epithelia of the intestine. Both PB22 and JCM20300 showed much less impact on the mucosa tissue, suggesting a negative correlation of the injury with tissue penetration. This injury induction of LGG accords with the higher expression of inflammatory cytokines shortly after treatment and stress-related Hsp70 after prolonged immersion in intestine of zebrafish treated with the mucosa type strains LP compared with LB and LR (Fig. 5). Therefore, the compromised protection activity of the well-colonized mucosa type strains was probably due to its injury to the mucosa of intestine, which counteracted the positive effect associated with greater tissue penetration. The injury of LGG to the intestine of zebrafish was a surprising result, as LGG has been reported to protect the intestinal epithelium from damage in mice^23,24^. This discrepancy might be due to: (i) the mucus of fish is much thinner than that of mammals^25^; and (ii) fish harbors inefficient adaptive immunity compared with mammals^26^, which may lead to weaker control of the commensal bacteria. Moreover, the strains used here were not isolated from the intestine of fish. Therefore, it cannot be ruled out that some indigenous *Lactobacillus* strains of fish may impact the host tissue differently after colonization, which deserves further study. Notably, the observed spatial distribution, as well as the correlation between spatial distribution and protection activity of *Lactobacillus*, cannot be directly extended to mammals, considering the discrepancy of multiple phenotypes observed in zebrafish relative to mice.

The SpaCBA pili play a key role for the adhesion of LGG to mucus^12,15^ and epithelial cells^16,17^. Consistently, the LGG mutant lacking SpaC showed significantly lower bacterial cells (by at least 2 orders of magnitude) adherent to murine intestinal inner surface^17^. However, our results revealed that mutant P22 lacking the SpaCBA pili exhibited the same level of overall colonization in the intestine of zebrafish compared with the wildtype control, suggesting a less important role of SpaCBA pili in the intestinal adhesion of LGG in zebrafish than in the case of mice. This discrepancy might be attributed to the difference in affinity of the LGG pili to the receptors on the intestinal inner surface of different hosts (zebrafish *vs* mice). The adhesion of LGG to both murine mucus and epithelial cells was a combined action mediated by multiple surface factors. Apart from the key role attributed to SpaCBA pili, other surface proteins have been reported to play a modulated or ancillary role in LGG adhesion to mucus or epithelial cells, such as mucus-binding factor (MBF)^27^ and modulator of adhesion and biofilm (MabA)^28^. Considering the minor contribution of SpaCBA pili in zebrafish, these factors or other unknown surface factors might play a more important role in the adhesion of LGG to the intestine of zebrafish. The impact of adhesion factors on the spatial distribution of *Lactobacillus* in the intestine of host has never been reported. In this study, the SpaCBA pili displayed an important role in the spatial distribution of LGG, as the mutant PB22 changed to hybrid type from the mucosa type of the wildtype strain. Presumably, the SpaCBA pili were an efficient mucosa adhesin in zebrafish, and its mutation impaired the distribution of LGG in the mucosa zone, while other factors were playing the key role in mucus adhesion, contributing to the overall unchanged colonization of PB22 in the intestine of zebrafish. The molecular mechanism underlying the spatial distribution of *Lactobacillus* awaits further investigation.

## Conclusion

Collectively, our data revealed a previously unreported correlation of spatial distribution of *Lactobacillus* with their protection activity in zebrafish. Further study on the molecular mechanisms underlying the spatial distribution and the correlation of distribution with disease protection will provide novel insights into host interactions of *Lactobacillus* and other commensal bacteria. Moreover, we observed that the mixture of mucus and mucosa type of *Lactobacillus* strains exhibited improved protection activity that is comparable to a hybrid type strain, which may guide the formulation of *Lactobacillus* probiotic products in the practice of aquaculture.

## Materials and Methods

### Experimental ethics

All experiments on zebrafish were performed according to the Chinese legislation associated with animal experimentation and the studies were approved by the Ethics Committee of the Feed Institute, Chinese Academy of Agricultural Sciences (2016-ZZG-ZF-02).

### Bacterial strains and culture condition

Of the 49 *Lactobacillus* strains tested in the present study, 45 strains are from the LAB collection centre, Inner Mongolia Agricultural University, China (Table S3). The bacterial strains used in our study are listed in Table S1. LGG mutant PB22 were obtained from the Department of Veterinary Biosciences, University of Helsinki, Helsinki, Finland. *A. hydrophila* strain NJ-1 was donated by College of Veterinary Medicine, Nanjing Agricultural University, Nanjing, China. All LABs were propagated The zebrafish were immersed in the* Lactobacillus* inoculated water for 14 days for 24 h in MRS medium; *A. hydrophila* NJ-1 was grown in tryptic soy broth (TSB, Difco Laboratories, Detroit, MI, USA) at 30 °C for 24 h.

### Experimental design

AB zebrafish about 2 months age were fed a standard diet (crude protein 42% and lipid 6.0%), and randomly divided into 10 L plastic tank with aerated fresh water. For *Lactobacillus* treatment, the tank water was added overnight culture of *Lactobacillus* strains (Tables S1 and S3) at a final concentration of 10^7^ CFU/ml. Each treatment had four replicates. The treated zebrafish were maintained under immersing *Lactobacillus* water for 14 days. About 3/4 volume of water from each tank was changed daily and the concentration of *Lactobacillus* was maintained by adding fresh culture of *Lactobacillus* after water change. The fish were handed fed twice time daily (9:30, 14:30). Standardized conditions were maintained: water was continuously mechanically and biologically filtered, aerated and kept at 28 ± 1 °C; pH, 7.5–7.8; Unionized Ammonia,<0.02 mg/ml; Nitrite,<0.1 mg/ml, DO >5.0 mg O/l; photoperiod was kept at 14:10 (light:dark cycle).

### Spatial distribution of the *Lactobacillus* strains in the intestine

After immersion in the *Lactobacillus*-inoculated water for 14 days, 8 fish from each treatment were randomly chosen. Fish were euthanized with 100 ppm of MS-222. The whole intestine was carefully dissected with sterile, fine-tipped forceps. The intestine was gently cut open and was added in 1 mL phosphate-buffered saline (PBS; 137 mM NaCl, 8 mM Na_2_HPO_4_, 3 mM KCl, and 1 mM KH_2_PO_4,_ pH 7.4). Then the intestine was agitated for 3 min at 250 rev min^−1^ by a vortex mixer IKA MS 3 basics (Wilmington, USA) to ensure the mucus were separated from the mucosa (Fig. S2). The gut wall was collected by filtration using a nylon mesh (100 μm). Thus, the mucus bacteria were in the filtrated supernatant while the gut wall sample contains the bacteria adhered in the mucosa zone. The gut wall was washed three times with PBS by centrifugation (3000 g) and resuspension, and were homogenized in 1 mL PBS. The filtrated supernatant and the homogenized gut wall were both subject to series 10 times dilutions and were cultured on MRS agar at 37 °C for 24 h for counting of the lactobacilli in the mucus and mucosa part, respectively.

### Sampling procedure

After immersion in *Lactobacillus* (LP, LB or LR), 6 fish from each treatment were randomly chosen at 5 h and 14 d. Fish were euthanized with 100 ppm of MS-222. The whole intestine was gently taken^21^. At each sampling point the dissected gut from 6 fish from each tank were stored at −70 °C for RT-PCR analysis. At day 14 post *Lactobacillus* immersion, extra 14 fish were randomly chosen from each treatment. 8 fish were used for analysis of the* Lactobacillus* spatial distribution; the other 6 fish were used for gut microbiota analysis.

### Quantitative real-time PCR

Total RNA from the gut was extracted using a TRIzon Reagent RNA kit according to the manufacturer’s instructions (Promega, Madison, WI). The reverse transcription (RT-PCR) was performed by using Rever Tra Ace-α-RT-PCR kit (TOYOBO, Shanghai, China) with an oligo (dT). qPCR cycling conditions: 10 min 95 at degrees Celsius, followed by 45 cycles of 30 sec at 95 degrees Celsius, 30 sec at 55 degrees Celsius, and 20 sec at 72 degrees Celsius. qPCR reactions were performed in triplicate and averaged. For each gene, gene expression levels were calculated relative to a reference gene, Rps1. The primers are listed in Table S2.

### Gut adhesive microbiota

To avoid inter-individual difference, gut samples of the 6 fish from each treatment were pooled together. Briefly, the total fish gut was sampled under sterile condition, then samples from 6 fish were pooled and homogenized in 1 ml PBS. Bacterial genomic DNA was extracted using a QIAamp DNA Stool Mini Kit (Qiagen, Germany) with slight modification. PCR was performed to target the V6–V8 region 968F-1401R of the 16 S rRNA gene^29^. The PCR reactions were performed three times, and the products of each sample were mixed together for preventing bias in amplification. The DNA concentration of the PCR product of each sample was measured by using a Quant-iT PicoGreen double-stranded DNA assay (Invitrogen, Germany), and their quality was controlled on an Agilent 2100 bioanalyzer (Agilent, USA). After quantitation, the amplicons from each reaction mixture were pooled in equimolar ratios and subjected to emulsion PCR to generate DNA library, as recommended by 454 Life Sciences. Amplicon pyrosequencing was performed from the A-end using a 454/Roche A sequencing primer kit on a Roche Genome Sequencer GS FLX Titanium platform at Majorbio Bio-Pharm Technology Co., Ltd., Shanghai, China.

Raw sequences obtained from the various amplicon pools were demultiplexed using the sff file utility from 454 Sequencing System Software (version 2.5.3) (454 LifeScience). In order to allow for a correct multiple alignment for ecology parameter evaluation we selected only the reads starting from the same side of the two amplicons. Specifically, reads starting from the 5′-end of the Bif and the 3′-end of the Puni were selected. The valid sequences were simplified using the ‘unique.seqs’ command to get a unique set of sequences, and then they were aligned using the ‘align.seqs’ command and compared with the Bacterial SILVA database. The aligned sequences were further trimmed and the redundant reads were eliminated using the ‘screen.seqs’, ‘filter.seqs’, and ‘unique.seqs’ commands in order. Then, the ‘dist.seqs’ command was performed, and unique sequences were clustered into operational taxonomic units (OTUs), defined at the 97% similarity threshold. Heatmap figure was generated using custom Perl scripts.

### Pathogen infection

*A. hydrophila* NJ-1 were grown in TSB media at 30 °C until stationary phase, then pelleted (7500 rpm for 10 min) and washed twice in sterile water. Bacteria were resuspended and transferred to separated tanks (3 L) at a final 10^8^ CFU/ml. Each tank was loaded with 10 fish, and triplicate tanks were used for each treatment group. 4 fish from each tank were chosen for detecting gut IAP 24 h post challenge. The challenged fish were kept under observation for 30 days and the mortalities were recorded.

### Intestinal Alkaline Phosphatase

Intestinal alkaline phosphatase (IAP) was estimated by using p-nitrophenyl phosphate as substrate by the method of Bates (2007)^30^.

### Histopathology

Six zebrafish from each group were sampled 14 days post *Lactobacillus* (LGG, PB22, or LR) immersion, the histopathologic change of the anterior, mid and posterior intestinal segments was observed by hematoxylin and eosin (H&E) staining. The intestine tissue samples were fixed with 4% paraformaldehyde overnight at 4 °C, dehydrated in a graded series of ethanol and xylene prior to embedding in paraffin wax. The specimens were cut into sections of 5 μm thickness, and subsequently stained with H&E. The structure of zebrafish intestine was observed under light microscope.

### Preparation of gnotobiotic zebrafish

GF zebrafish were prepared following established protocols^31^with some modifications. Briefly, embryos 6 h post-fertilization (hpf) were soaked in 0.1% polyvinylpyrrolidoneiodine (Sigma) for 2 min and washed 3 times in gnotobiotic zebrafish media (GZM). Thereafter, the embryos were further soaked in 0.003% bleach for 10 min and washed by GZM. Lastly, GF embryos were transferred to a 25-cm^2^ cell culture flask (Nest Biotechnology Co.) containing 30 mL of sterile GZM.

### Direct treatment of GF zebrafish with *Lactobacillus* strains

Gnotobiotic larvae at 3 dpf were immersed in a final concentration of fresh culture of LGG or PB22 at 10^7^ CFUs/mL. At 6 dpf, the zebrafish were challenged with *A. hydrophila* NJ-1 as described above.

### Transfer of gut bacteria from adult zebrafish to GF recipients

The gut of fish treated with or without *Lactobacillus* (LGG, PB22) for 2 wk were collected. Briefly, the gut samples from 6 fish were pooled and homogenized in 1 mL PBS. The intestine homogenate was centrifuged at 2000 g to remove particles. Then the bacterial suspension was added to GZM containing 3 dpf gnotobiotic larvae at a final concentration of 10^6^, 10^5^ and 10^4^ CFU/mL of GZM. The concentration of bacteria in GZM was confirmed by culture on lysogeny broth (aerobic) and brain-heart infusion-blood (anaerobic) agar for 24 h at 30 °C. At 6 dpf, the zebrafish were challenged with *A. hydrophila* NJ-1 as described above. The transfer efficacy was confirmed by DGGE as described in previous study^22^.

### Statistical Analysis

Data are expressed as the mean ± SEM. Unpaired Student’s *t*-test and ANOVA were used to analyze the data. Significant differences were accepted at *P < *0.05.

## Electronic supplementary material

Supplementary Information
