# Supplementary material for: Anti-Infective Effect of Adhesive Probiotic Lactobacillus in Fish is Correlated With Their Spatial Distribution in the Intestinal Tissue
Source: Sci Rep. 2017 Oct 16;7:13195. doi: 10.1038/s41598-017-13466-1 (PMC5643340; doi:10.1038/s41598-017-13466-1)
Supplement: Supplementary file 1 — Supplementary Information [file 41598_2017_13466_MOESM1_ESM.doc]

**Anti-Infective Effect of Adhesive Probiotic *Lactobacillus* in Fish is Correlated With Their Spatial Distribution in the Intestinal Tissue**

Suxu He^1^, Chao Ran^1^, Chubin Qin^1^, Shuning Li^1^, Hongling Zhang^1^, Willem M. de Vos^2^, Einar Ringø^3^, Zhigang Zhou^1*^

^1^Key Laboratory for Feed Biotechnology of the Ministry of Agriculture, Feed Research Institute, Chinese Academy of Agricultural Sciences, Beijing 100081, P. R. China.^2^Laboratory of Microbiology, Wageningen University, Wageningen, Netherlands and Departments of Bacteriology & Immunology and Veterinary Biosciences, University of Helsinki, Helsinki, Finland.^3^Norwegian College of Fishery Science, Faculty of Bioscience, Fisheries and Economics, University of Tromsø, NO-9037 Tromsø, Norway

Correspondence and requests for materials should be addressed to Z.Z. (email: [zhouzhigang03@caas.cn](mailto:zhouzhigang03@caas.cn))

**Supplemental data**

### Pathogen growth inhibition assays. *Lactobacillus* strains were grown 18–24 h anaerobically at 37°C in MRS broth. Two microlitre (2 μl)-droplets of standardized culture broth containing approximately 10^9^ cells ml^−1^ (as estimated by OD_600_ absorbance readings) were spotted onto MRS agar and incubated anaerobically for 24 h at 37°C. The pathogen *Aeromonas hydrophila* NJ-1 was grown aerobically in brain–heart infusion (BHI) broth for 24 h at 30°C and used to inoculate 10 ml soft BHI agar (0·7% agar; molten and tempered to 45°C) to obtain standardized bacterial suspensions of 10^7^ cells per plate. Plates were carefully overlaid with suspensions and incubated aerobically for 24 h at 30°C to obtain bacterial lawns. The radius of pathogen growth inhibition zone was measured in milimetres. Assays were performed twice, both in triplicate.

### Long-term survival experiment in water. Each bacterial suspension was dispensed into a 3 L tanks to dose at 10^8^ cfu/ml. Samples were taken at 0, 12, 24, 36 and 48 h, respectively. Viable cell counts were determined by pour-plating the appropriate dilution with MRS agar. The plates were incubated at 37 °C for 48 h.

### *Lactobacillus* bath dose trial. The zebrafish were bathed with different doses (10^4^, 10^5^, 10^6^ and 10^7^cfu /ml) of *Lactobacillus* strains for 14 days, with the fresh culture of *Lactobacillus* strains exchanged each day. 6 fish from each treatment were chosen at day 3, 7 and 14. The gut was sampled and the colonized LABS were estimated by further serial 10-fold dilutions in PBS and spread plate method on MRS agar.

### Alcian blue staining method for mucus separation assay. The intestine was gently cut open and was added in 1 mL phosphate-buffered saline. Then the intestine was agitated at 250 rev min^−1^ by a vortex mixer IKA MS 3 basics (Wilmington, USA), and the gut were sampled at the 0, 90 and 180s to test when the mucus were totally separated from the mucosa. The treated intestine were put in 1% Alcian blue pH 2.5 (Sigma-Aldrich) for 5 min, followed by differentiation in 0.37%HCl/70%EtOH for 30 min. After washing in water, the intestines were observed with microscope.

**Colonization dynamic of LABs in gut**. The fish were bathed in 10^7^cfu/ml of a *Lactobacillus* strain for 14 days, then transferred to fresh water without *Lactobacillus*. After 0, 6, 12, 24, 36 and 48h, 6 fish were chosen, and the mucus- and mucosa-colonized *Lactobacillus* were distinguished as described in the main text. The bacterial suspension was 10-fold diluted in PBS and cultured on MRS agar at 37 °C .

**Bacterial Enumeration in gut.** Homogenized mucus and gut wall were serially diluted in PBS. Then, aliquots of the appropriate dilutions were spread onto Brain Heart Infusion (BHI; Difco, Detroit, MI) followed by overnight incubation under anaerobic conditions at 30°C for 24h. Aerobes were cultured with the nonselective agar standard-plate-count agar (Difco) at 30°C for 24h.

**Figure S1. Specific characteric of LABs .** (A) In vitro antagonistic activity of the three *Lactobacillus* strains against *A. hydrophil*a NJ-1 strain. (B) The survival of the three strains in [recycled](javascript:void(0);) [water](javascript:void(0);) at 28℃. (C) Colonization kinetics of the three strains in zebrafish sampled at day 3, 7 and 14.

Each of the values is the mean of three replicates. Values followed by different letters are significantly different (*p* < 0.05).


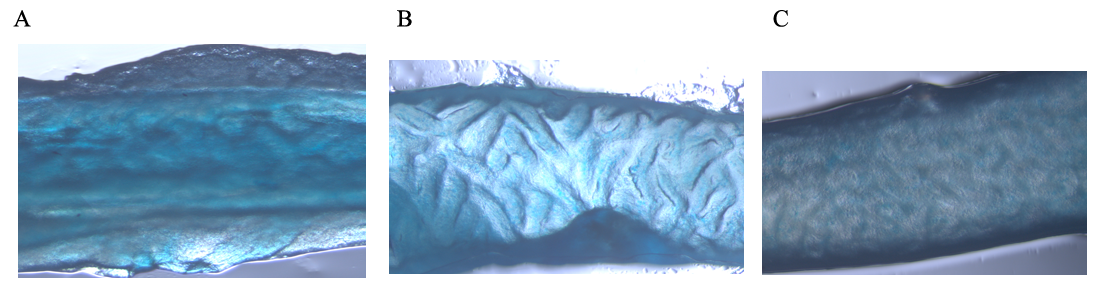


**Figure S2. Confirmation of mucus separation by Alcian blue staining.** (A) The intestinal mucus stained with Alcian blue (B) The intestine mucus after 90s vortexing treatment. (C) The intestine mucus after 180 s vortexing treatment. After vortexing at 250 rev min^−1^ for 180s, there is no mucus left in the intestine.

**Figure S3. The level of each strain adhered in mucus and mucosa of zebrafish gut after continuous bath for 14 days**. (A) The level of each strain in mucosa. (B) The level of each strain in mucus. Data are representative of two independent experiments. n=10 fish.

**Figure S4. The releasing dynamics of the three *Lactobacillus* strains in gut of zebrafish after cessation of immersion treatment.** (A) Numbers of LP CFUs in the gut wall sampled at 0, 6, 12, 24 , 36 and 48 h.(B) Numbers of LP CFUs in the mucus sampled at 0, 6, 12, 24 , 36 and 48 h. (C) Numbers of LB CFUs in the gut wall sampled at 0, 6, 12, 24 , 36 and 48 h. (D) Numbers of LB CFUs in the mucus sampled at 0, 6, 12, 24 , 36 and 48 h. (E) Numbers of LR CFUs in the gut wall sampled at 0, 6, 12, 24 , 36 and 48 h. (F) Numbers of LR CFUs in the mucus sampled at 0, 6, 12, 24 , 36 and 48 h. N=6 , Mean values and SEM are plotted.

**Fig S5** **Spatial distribution and disease protection activity of LGG and LGG-PB12 in zebrafish**. (A) The overall colonization level of LGG or PB12 in the fish gut at 10^6^ cfu/ml immersion. (B) Spatial distribution of LGG or PB12 at 10^6^ cfu/ml immersion. (C) The overall colonization level of LGG or PB12 in the fish gut at 10^7^ cfu/ml immersion. (**D**) Spatial distribution of LGG or PB12 at 10^7^ cfu/ml immersion. (E) Cumulative survival of LGG or PB12 treated zebrafish after *A. hydrophila* NJ-1 infection. LGG-PB12 is pilus deficient due to a mutation of the *srtC1* gene and it has a total of 24 other SNPs.

**Figure S6. The total amount of selected *Lactobacillus* strains colonized in the intestine of zebrafish after continuous immersion for 14 days**. n=8 fish/group.

**Table S1 The strains used in the study**

| Strain name | [Abbreviation](javascript:void(0);) | Description |
| --- | --- | --- |
| *Lactobacillus plantarum* JCM 1149^T^ | LP | Probiotics, culture in MRS at 37℃ |
| *Lactobacillus brevis* JCM 1170 | LB | Probiotics, culture in MRS at 37℃ |
| *Lactobacillus rhamnosus* JCM 20300 | LR | Probiotics, culture in MRS at 37℃ |
| *Lactobacillus rhamnosus* ATCC 53103 | LGG | Probiotics, culture in MRS at 37℃ |
| L. rhamnosus LGG- PB22 | PB22 | pilus deficient as it has lost the pilus island and flanking sequences (75 kb DNA) and has 51 other SNPs |
| *Aeromonas hydrophila* NJ-1 | NJ-1 | Aquaculture pathogen of common carp, culture in TSB at 30℃ |

**Table S 2 Primers for real time PCR analysis.**

| Gene |  | sequence | Accession NO. |
| --- | --- | --- | --- |
| IL-1β | F | CATCAAACCCCAATCCACAG | [NM_212844.2](http://www.ncbi.nlm.nih.gov/nuccore/NM_212844.2) |
|  | R | CACCACGTTCACTTCACGCT |  |
| tnfa | F | GCGCTTTTCTGAATCCTACG | NM_212859 |
|  | R | GCCCAGTCTGTCTCCTTCT |  |
| TGF-β | F | GTCGCTGCATTGAAACAGAA | [XM_001342570.6](http://www.ncbi.nlm.nih.gov/nuccore/XM_001342570.6) |
|  | R | CTTAACCCATGGAGCAGAGG |  |
| Hsp70 | F | CTGGACTGAATGTTGCTCGC | [NM_001113589.1](http://www.ncbi.nlm.nih.gov/nuccore/NM_001113589.1) |
|  | R | CAGATGAGTGTCTCCAGCGG |  |
| Saa | F | CGCAGAGGCAATTCAGAT | [NM_001005599.1](http://www.ncbi.nlm.nih.gov/nuccore/NM_001005599.1) |
|  | R | CAGGCCTTTAAGTCTGTATTTGTTG |  |
| Cfb | F | GCCACAGTGCTACGCTGATTT | [NM_131338.2](http://www.ncbi.nlm.nih.gov/nuccore/NM_131338.2) |
|  | R | GTTGAACTGTTAGAGTTGTCGTTAGAGAATT |  |
| rps11 | F | ACAGAAATGCCCCTTCACTG | NM_213377.1 |
|  | R | GCCTCTTCTCAAAACGGTTG |  |

**Table S3 The 49 *Lactobacillus* strains message used in our study**.

| **No.** | **Strain name** | **Short name** | **16SGenBank** |
| --- | --- | --- | --- |
| 1 | *Lactobacillus plantarum NM104-2* | NM104-2 | HM218444 |
| 2 | *Lactobacillus fermentum G27-4* | G27-4 | HM058798 |
| 3 | *Lactobacillus reuteri NM96-5* | NM96-5 | HM218407 |
| 4 | *Lactobacillus plantarum S23-3* | S23-3 | HM058605 |
| 5 | *Lactobacillus fermentum MGA39-4* | MGA39-4 | HM057958 |
| 6 | *Lactobacillus delbrueckii subsp. Bulgaricus MGD9-2* | MGD9-2 | HM058500 |
| 7 | *Lactobacillus plantarum NM102-1* | NM102-1 | HM218429 |
| 8 | *Lactobacillus casei NM103-7* | NM103-7 | HM218442 |
| 9 | *Lactobacillus casei NM105-4* | NM105-4 | HM218453 |
| 10 | *Lactobacillus acidophilus D1301-1-1* | D1301-1-1 | EF749655 |
| 11 | *Lactobacillus plantarum G72-2* | G72-2 | HM058972 |
| 12 | *Lactobacillus casei NM103-6* | NM103-6 | HM218441 |
| 13 | *Lactobacillus delbrueckii subsp. Bulgaricus MGA17-3* | MGA17-3 | HM057871 |
| 14 | *Lactobacillus plantarum NM66-1* | NM66-1 | HM218295 |
| 15 | *Lactobacillus fermentum YN35-1-1-1* | YN35-1-1-1 | FJ749485 |
| 16 | *Lactobacillus reuteri NM 72-4* | NM72-4 | HM218331 |
| 17 | *Lactobacillus fermentum G19-5* | G19-5 | HM058765 |
| 18 | *Lactobacillus fermentum YN3-1-1-2* | YN3-1-1-2 | FJ749470 |
| 19 | *Lactobacillus plantarum NM65-5* | NM65-5 | HM218293 |
| 20 | *Lactobacillus plantarum QH30-1* | QH30-1 | FJ749345 |
| 21 | *Lactobacillus plantarum BM1305* | BM1305 | GQ131122 |
| 22 | *Lactobacillus fermentum MGA22-4* | MGA22-4 | HM057897 |
| 23 | *Lactobacillus plantarum QH14-2-2* | QH14-2-2 | FJ749733 |
| 24 | *Lactobacillus plantarum BX6-2* | BX6-2 | FJ749576 |
| 25 | *Lactobacillus reuteri HS1302* | HS1302 | GQ131183 |
| 26 | *Lactobacillus reuteri NM93-1* | NM93-1 | HM218386 |
| 27 | *Lactobacillus casei G54-7* | G54-7 | HM058908 |
| 28 | *Lactobacillus casei NM13-4* | NM13-4 | HM218068 |
| 29 | *Lactobacillus acidophilus HS8-2* | HS8-2 | JQ805676 |
| 30 | *Lactobacillus fermentum NM98-5* | NM98-5 | HM218414 |
| 31 | *Lactobacillus casei NM11-5* | NM11-5 | HM218059 |
| 32 | *Lactobacillus reuteri DSM20016* | DSM20016 | NC_009513.1 |
| 33 | *Lactobacillus fermentum S17-7* | S17-7 | HM058584 |
| 34 | *Lactobacillus plantarum QH46-5-1-1* | QH46-5-1-1 | FJ749395 |
| 35 | *Lactobacillus delbrueckii subsp. Bulgaricus MGA17-6* | MGA17-6 | HM057874 |
| 36 | *Lactobacillus fermentum NM97-4* | NM97-4 | HM218410 |
| 37 | *Lactobacillus fermentum G73-4* | G73-4 | HM058978 |
| 38 | *Lactobacillus reuteri NM98-1* | NM98-1 | HM218411 |
| 39 | *Lactobacillus casei NM8-1* | NM8-1 | HM218042 |
| 40 | *Lactobacillus plantarumQH14-8* | QH14-8 | FJ749722 |
| 41 | *Lactobacillus reuteri NM95-6* | NM95-6 | HM218402 |
| 42 | *Lactobacillus casei NM102-4* | NM102-4 | HM218432 |
| 43 | *Lactobacillus casei WMGB84-5* | WMGB84-5 | HM058288 |
| 44 | *Lactobacillus casei AG8-5* | AG8-5 | FJ749569 |
| 45 | *Lactobacillus casei NM26-7* | NM26-7 | HM218133 |
| 46 | *Lactobacillus plantarum JCM 1149T* | JCM 1149T | NR_115605.1 |
| 47 | *Lactobacillus brevis JCM 1170* | JCM 1170 | NC_008497.1 |
| 48 | *Lactobacillus Rhamnosus JCM 20300* | JCM 20300 | JF414108 |
| 49 | *Lactobacillus rhamnosus ATCC 53103* | ATCC 53103 | NC_013198.1 |
